# In situ IR spectroscopy data and effect of the operational parameters on the photocatalytic activity of N-doped TiO_2_

**DOI:** 10.1016/j.dib.2019.103917

**Published:** 2019-04-28

**Authors:** N.S. Kovalevskiy, S.A. Selishcheva, M.I. Solovyeva, D.S. Selishchev

**Affiliations:** a Novosibirsk State University, Pirogova 2, Novosibirsk, 630090, Russia; b Boreskov Institute of Catalysis, Lavrentieva 5, Novosibirsk, 630090, Russia

**Keywords:** TiO_2_ photocatalysis, Photocatalytic oxidation, N-doped TiO_2_, UV light, Visible light, FTIR spectroscopy, Continuous-flow set-up, Steady-state oxidation rate, Optimal parameters

## Abstract

The TiO_2_ photocatalyst doped with nitrogen was synthesized via a precipitation method and investigated in the oxidation of acetone vapor under UV (371 nm) and visible light (450 nm). The data were collected in a continuous-flow set-up equipped with a long-path IR gas cell for *in situ* analysis of oxidation products and evaluation of the photocatalytic activity. The IR spectra for inlet and outlet reaction mixtures and their change during the process are presented. A technique for quantitative analysis of initial substrate and oxidation product using collected IR spectra is described. The effects of main operational parameters, namely, outlet concentration of oxidizing substrate in the range of 0–25 μmol/L, humidity in the range of 10–85%, and surface density of photocatalyst in the range of 0.6–5.7 mg/cm^2^ were investigated, and the data received are presented. The data show the influence of these parameters on the UV and visible light photocatalytic activity of N-doped TiO_2_. The data is publicly available on GitHub according to the link: https://github.com/1kovalevskiy/Effect-of-the-operational-parameters.

**Table:** Specifications table

Subject area	Chemistry
More specific subject area	Photocatalysis
Type of data	Figure
How data was acquired	A continuous-flow set-up equipped with a special valve system for analysis of the inlet and outlet reaction mixtures using IR spectroscopy. IR spectroscopy: an FTIR spectrometer FT-801 from Simex LLC (Russia) equipped with a long-path IR gas cell (Infrared Analysis Inc., USA)
Data format	Raw and analyzed
Experimental factors	N-doped TiO_2_ photocatalyst was prepared via a precipitation method using titanyl sulfate as a titanium precursor and ammonium hydroxide as a precipitating agent, as well as a source of nitrogen. Before photocatalytic experiments, the synthesized photocatalyst was deposited on a 9 cm^2^ glass plate from an aqueous suspension followed by drying in air at 110 °C
Experimental features	The synthesized photocatalyst was tested in the oxidation of acetone vapor under UV (371 nm) and visible light (450 nm) in the continuous-flow set-up under steady-state conditions. Acetone was selected as a test organic substrate due to a fact that it does not cause the deactivation of photocatalyst and is completely oxidized to CO_2_ and water without gaseous intermediates. The effects of acetone concentration, humidity, and surface density on the UV and visible light photocatalytic activity of N-doped TiO_2_ were studied
Data source location	Novosibirsk State University, Pirogova 2, Novosibirsk 630090, Russian Federation
Data accessibility	Data is publicly available on GitHub (https://github.com/1kovalevskiy/Effect-of-the-operational-parameters)
Related research article	T.N. Filippov, D.A. Svintsitskiy, I.A. Chetyrin, I.P. Prosvirin, D.S. Selishchev, D.V. Kozlov, Photocatalytic and photochemical processes on the surface of uranyl-modified oxides: An *in situ* XPS study, Appl. Catal. A Gen. 558 (2018) 81–90 [1]

**Table:** 

**Value of the data** • Data allow for comparing the efficiency of the photocatalytic oxidation using N-doped TiO2 under UV and visible light • Data are useful for selection of the optimal parameters to compare different photocatalytic materials • In situ IR spectroscopy has great promise for the investigation of photocatalytic activity in the oxidation of volatile organic compounds • Data show great promise of continuous-flow set-ups for the investigation of kinetic characteristics and stability of photocatalysts

## 1 Data

**Fig. 1:**
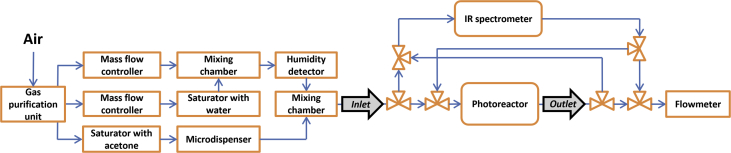
Schematic diagram of continuous-flow set-up used for the photocatalytic experiments.

**Fig. 2:**
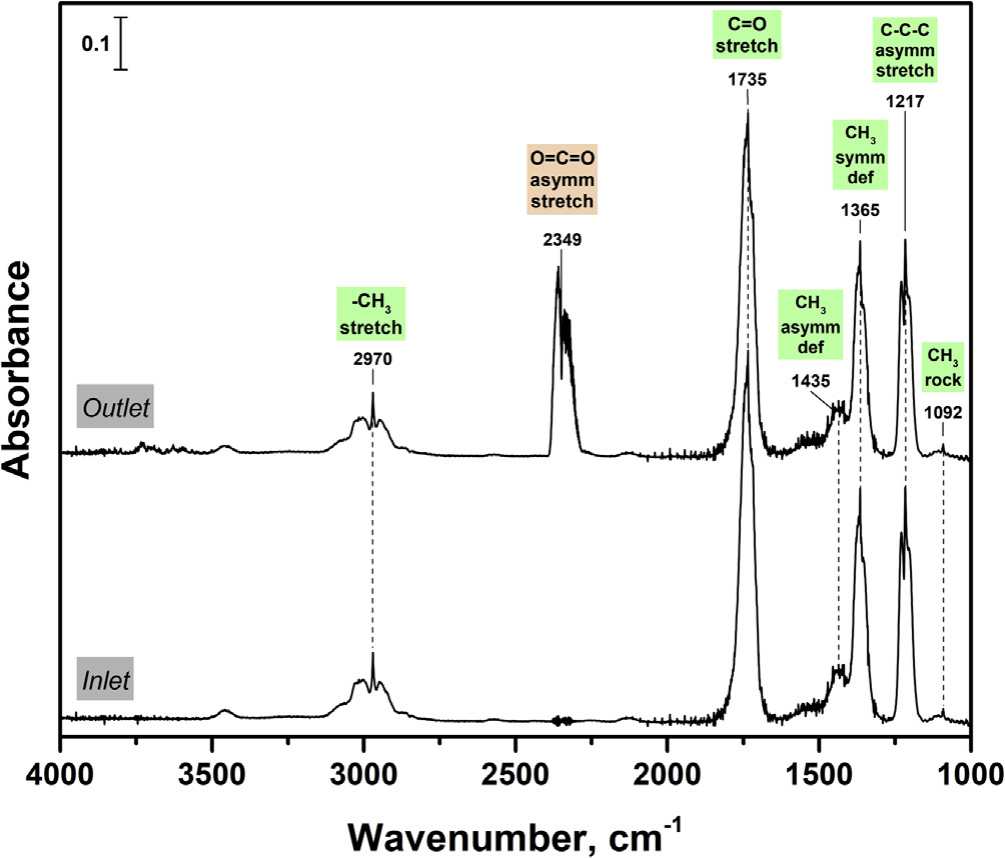
Typical IR spectra for inlet and outlet reaction mixtures during the photocatalytic oxidation of acetone vapor.

**Fig. 3:**
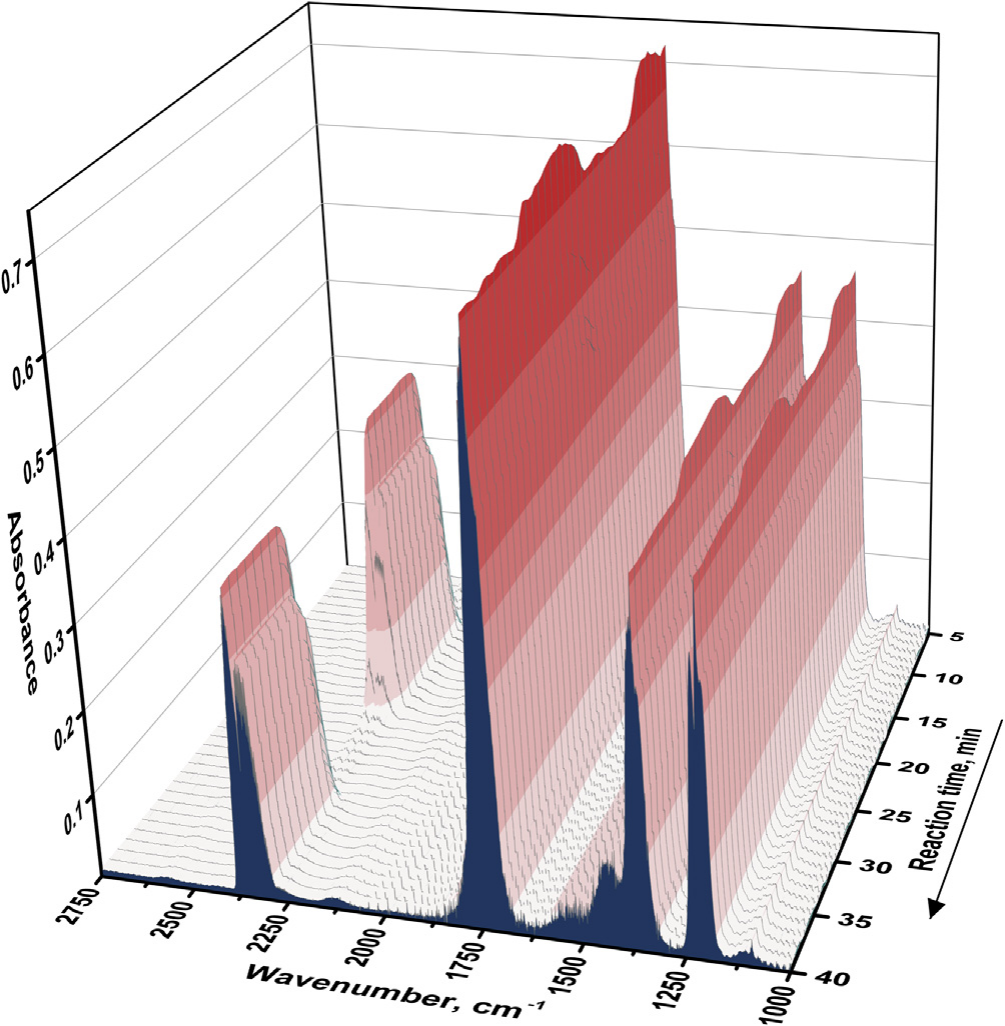
Evolution of the IR spectra during the experiment of acetone PCO.

**Fig. 4:**
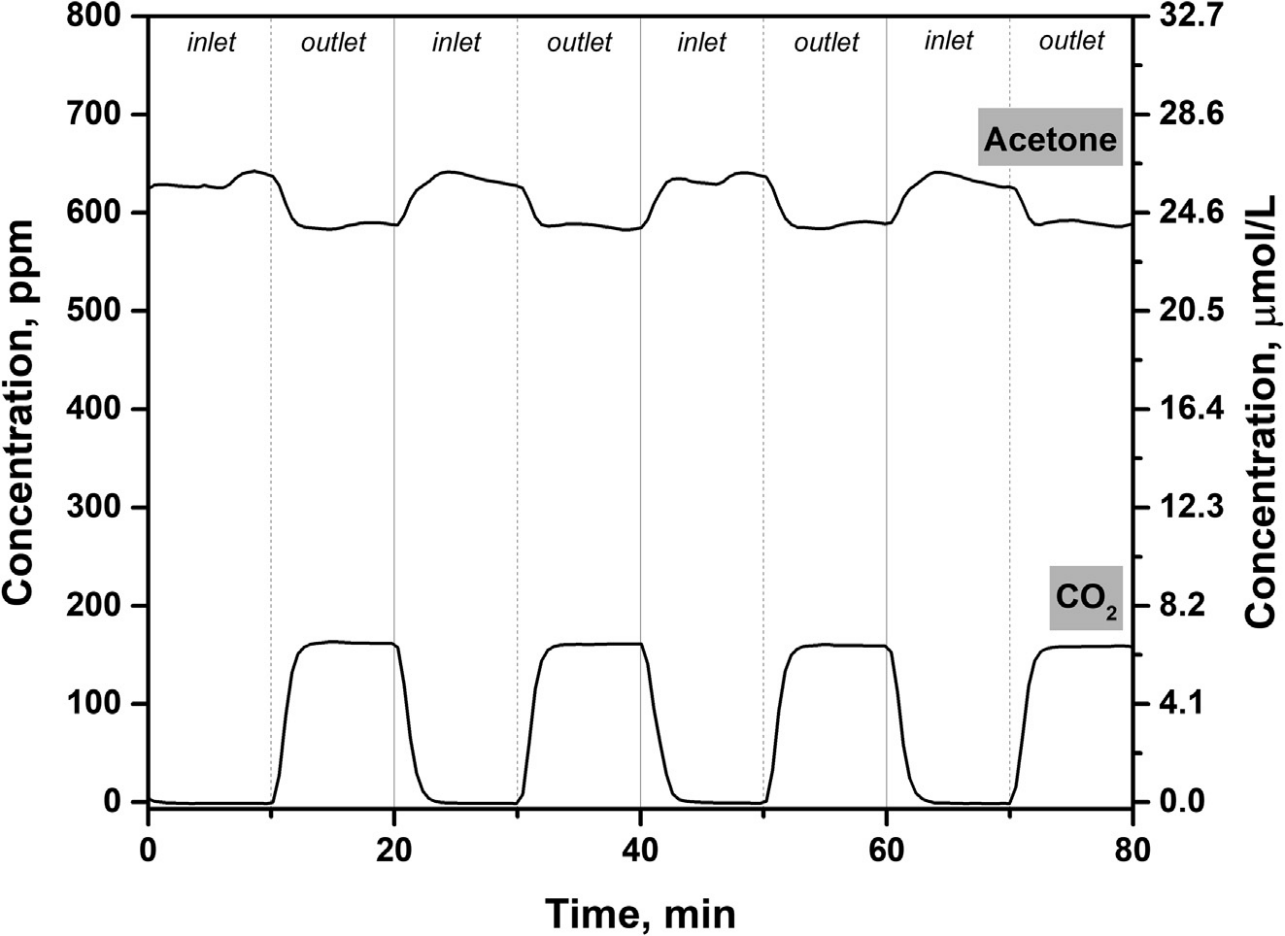
Acetone and CO_2_ concentration profiles during the experiment of acetone PCO.

The TiO_2_ photocatalyst doped with nitrogen was tested in a continuous-flow set-up during the oxidation of acetone vapor under UV (371 nm) and visible light (450 nm) to receive the data on the effects of operational parameters on the steady-state photocatalytic activity. The schematic diagram of the experimental set-up is shown in Fig. 1. A qualitative and quantitative analysis during the photocatalytic oxidation (PCO) process was performed using *in situ* IR spectroscopy technique. Fig. 2 shows the typical IR spectra for inlet and outlet reaction mixtures during the photocatalytic oxidation of acetone vapor. The IR spectra were collected periodically every 30 s to monitor the concentrations of acetone and CO_2_ during the PCO experiment. To illustrate this point, Fig. 3 shows the evolution of IR spectra during an experiment of acetone PCO with two switching between monitoring of inlet and outlet mixtures for 10 minutes. The IR spectra collected were analyzed, and the concentrations for acetone and CO_2_ were estimated according to the Beer-Lambert law. Fig. 4 shows the typical acetone and CO_2_ concentration profiles during the PCO experiment.

**Fig. 5:**
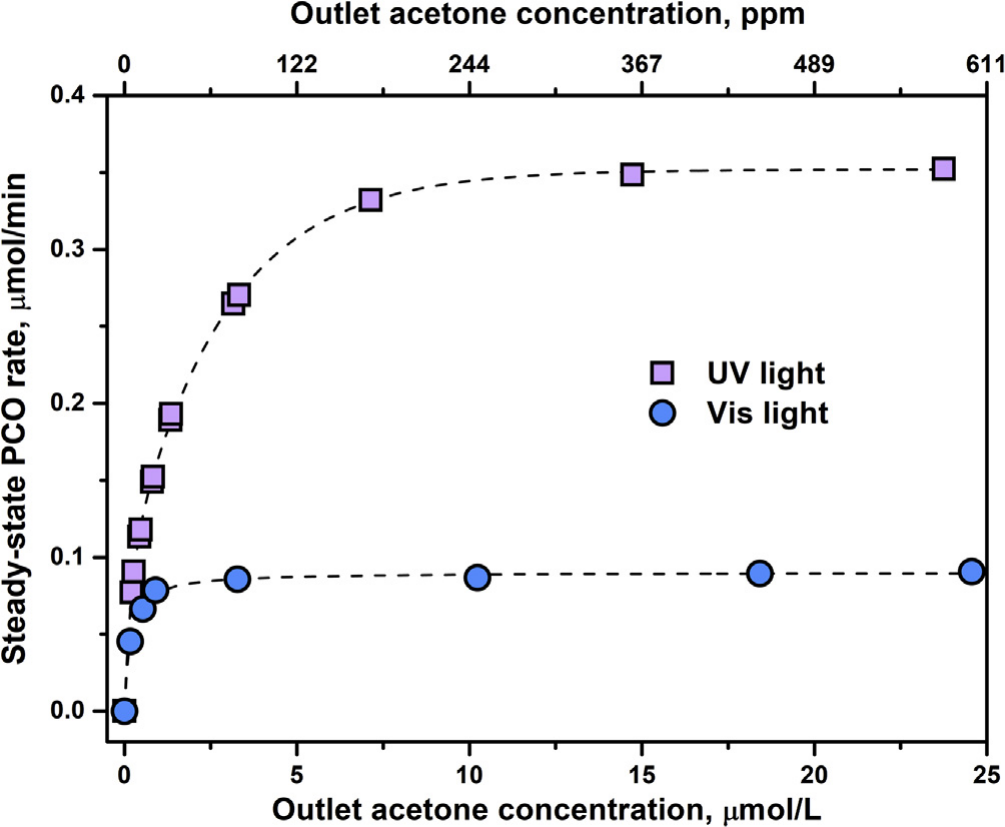
Effect of the outlet acetone concentration on the photocatalytic activity under UV and visible light.

**Fig. 6:**
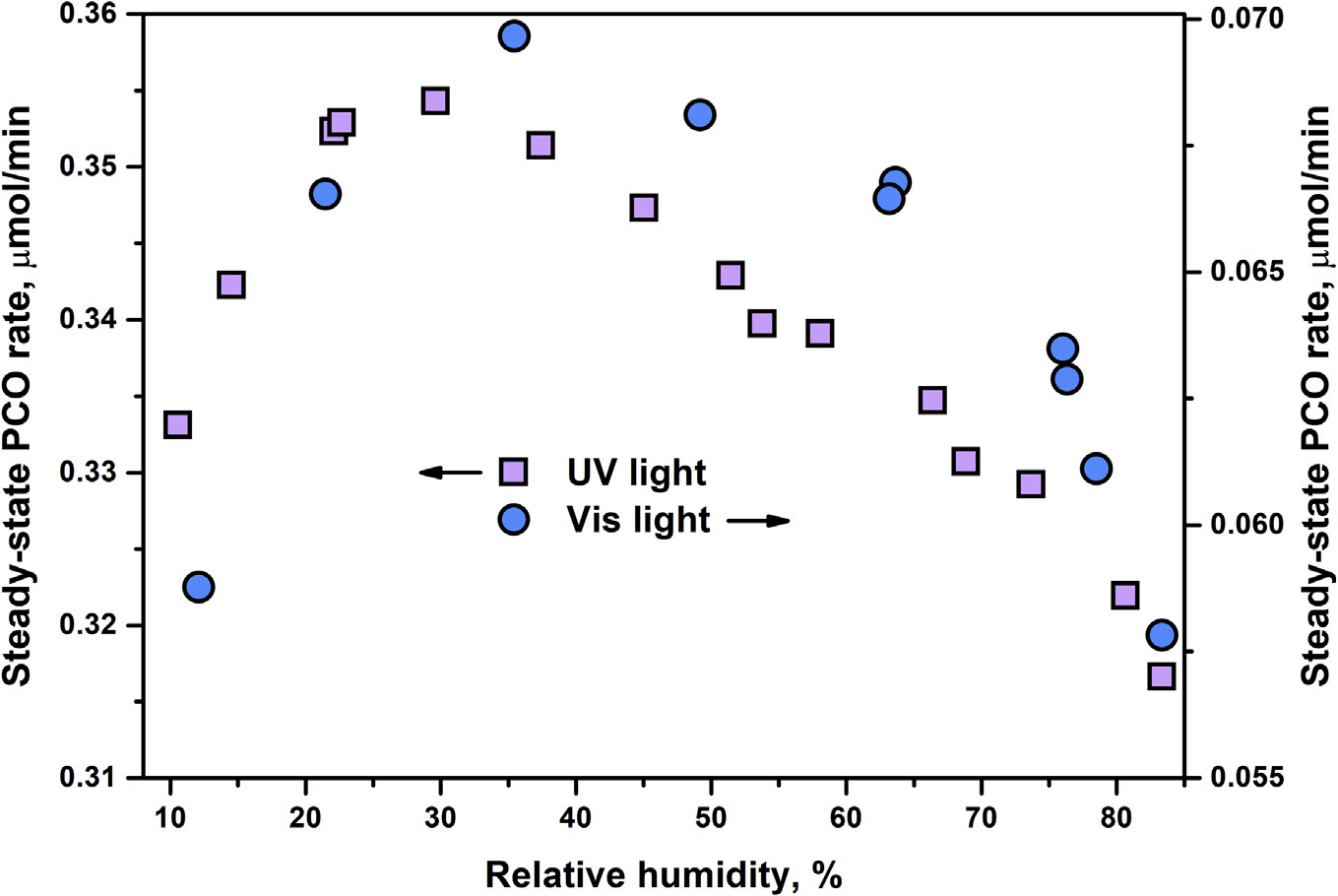
Effect of the relative humidity on the photocatalytic activity under UV and visible light.

**Fig. 7:**
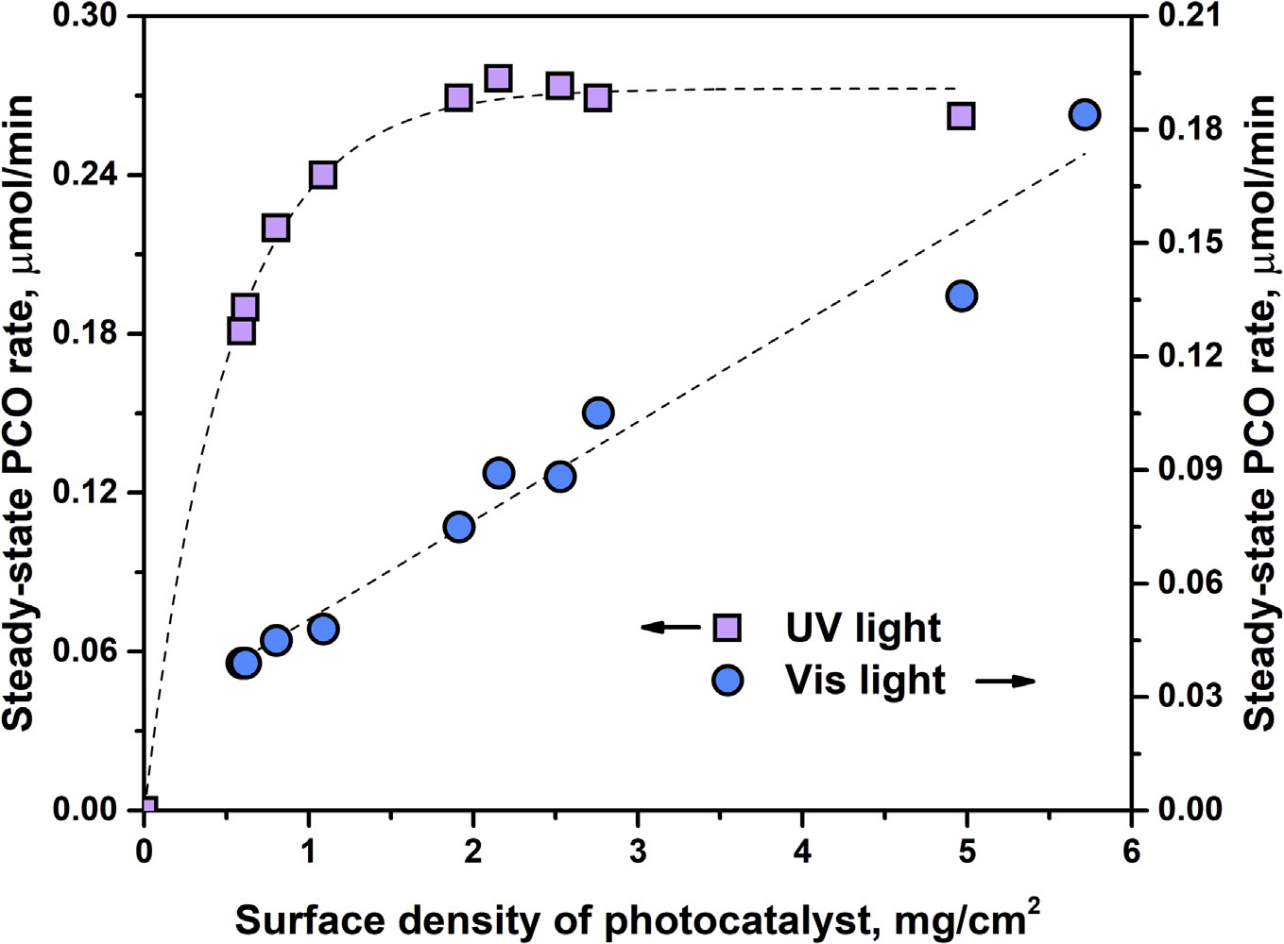
Effect of the photocatalyst surface density on the photocatalytic activity of N-doped TiO_2_ under UV and visible light.

The effects of main operational parameters, namely, outlet concentration of oxidizing substrate in the range of 0–25 μmol/L, humidity in the range of 10–85%, and surface density of photocatalyst in the range of 0.6–5.7 mg/cm^2^ were investigated, and the data received are presented. Fig. 5 shows the dependence of steady-state PCO rate for N-doped TiO_2_ under UV and visible light on the concentration of acetone in the outlet reaction mixture. Fig. 6 shows the effect of relative humidity on the photocatalytic activity of N-doped TiO_2_ under UV and visible light. Fig. 7 shows the dependence of steady-state PCO rate under UV and visible light on the photocatalyst surface density.

## 2 Experimental design, materials, and methods

### 2.1 Experimental set-up

The TiO_2_ photocatalyst doped with nitrogen was tested in the oxidation of acetone vapor under UV and visible light in the continuous-flow set-up to determine the steady-state photocatalytic activity and to investigate the effect of operational parameters on the activity. This continuous-flow set-up was previously successfully employed for the investigation of various photocatalytic materials and target pollutants [1], [2], [3], [4]. The set-up had the gas purification unit to remove particles, CO_2_, water vapor, and volatile organic compounds traces from air. The purified air flow was divided into three flows. Two flows were saturated with water and acetone vapor, respectively. Then, all the flows were mixed. The total volume rate, humidity, and concentration of acetone vapor in the reaction mixture was adjusted by the rate for each flow. The N-doped TiO_2_ photocatalyst was prepared via a precipitation method using titanyl sulfate as a titanium precursor and ammonium hydroxide as a precipitating agent, as well as a source of nitrogen. The synthesized photocatalyst was deposited on a 9 cm^2^ glass plate from an aqueous suspension and placed into the photoreactor. The surface density of photocatalyst on the glass plate was varied from 0.6 to 5.7 mg/cm^2^. The set-up had a special valve system that allows for analyzing the inlet and outlet reaction mixtures alternately using an FTIR spectrometer FT-801 from Simex LLC (Russia) equipped with a long-path IR gas cell (Infrared Analysis Inc., USA). During the analysis of inlet (10 min), the gas from a mixing chamber flows firstly through the IR cell and then goes to the photoreactor. In the case of outlet analysis (10 min), the gas flows through the photoreactor and then through the IR cell. The other experimental parameters were as follows: the reactor temperature is 40.0 ± 0.1°С, the volume flow rate is 0.069 ± 0.001 L/min.

### 2.2 IR spectroscopy analysis

As stated above, the special valve system allows for analyzing the inlet and outlet reaction mixtures alternately using IR spectroscopy. According to the NIST database [5], the bands at 1092, 1217, 1365, 1435, 1735, and 2970 cm^−1^ can be attributed to the acetone molecule that is the initial oxidizing substrate. In addition to these bands, the band at 2349 cm^−1^ was appeared in the IR spectra that correspond to the outlet mixture. This band can be attributed to CO_2_ molecule [5]. No other carbon-containing compounds were detected using IR spectroscopy. This result indicates that CO_2_ is the major product during the acetone PCO over N-doped TiO_2_ both under UV and visible light.

The IR spectra were collected periodically every 30 s to monitor the concentrations of acetone and CO_2_ during the experiment. The quantitative analysis was performed by the integration of collected IR spectra using the Beer-Lambert law as follows:

(1) $$\int_{\omega_{1}}^{\omega_{2}}A\left(\omega \right)d\omega =\varepsilon \times 1\times C$$

where $A\left(\omega \right)=\mathrm{lg}(I_{0}\left(\omega \right)/I\left(\omega \right))$ is the absorbance, $\omega_{1}$ and $\omega_{2}$ are the limits of the corresponding absorption bands (cm^−1^), ε is the attenuation coefficient (L/(μmol·cm^2^)), l is the optical path length (cm), and C is the concentration of a substance in the gas phase (μmol/L). The regions for the integration were selected as follows: 1160–1263 cm^−1^ for acetone and 2230–2450 cm^−1^ for CO_2_. The attenuation coefficients for each substrate were calculated from the calibration data. The regions for other compounds, which may be detected as intermediates during the PCO process, can be found elsewhere [6], [7], [8], [9].

### 2.3 Photocatalytic activity

Before the photocatalytic test, the adsorption-desorption equilibrium of acetone on the photocatalyst was achieved until no difference in inlet and outlet acetone concentrations was observed. After that, UV or visible light source was turned on and the photocatalytic activity was evaluated. A high-power UV-LED with a maximum at 371 nm and Vis-LED with a maximum at 450 nm were used for the photocatalyst irradiation. The total irradiance was 9.7 mW/cm^2^ for UV-LED and 145 mW/cm^2^ for Vis-LED. The photocatalytic activity was estimated as the steady-state PCO rate of the acetone oxidation. The PCO rate can be expressed as follows:

(2) $$\text{PCO}\;\text{rate}=\frac{\Delta C_{CO_{2}}\times U}{3}$$

where PCO rate is the steady-state photocatalytic oxidation rate (μmol/min), $\Delta C_{CO_{2}}$ is the difference in the outlet and inlet CO_2_ concentration (μmol/L), U is the volume flow rate (L/min). Typically, the CO_2_ concentration in the outlet increases as the irradiation time increases until a constant value that corresponds to the achievement of a steady state. The time required for the achievement of steady state depended on the activity of the catalyst and its adsorption capacity. The data for CO_2_ concentration from the region, which corresponds to the steady state, were used for the calculation of PCO rate. Based on the statistics of many experiments, a total error in measuring the PCO rate using the set-up does not exceed 10%.
