# Supplementary material for: In situ IR spectroscopy data and effect of the operational parameters on the photocatalytic activity of N-doped TiO2
Source: Data Brief. 2019 Apr 28;24:103917. doi: 10.1016/j.dib.2019.103917 (PMC6517577; doi:10.1016/j.dib.2019.103917)
Supplement: Multimedia component 1 [file mmc1.docx]

Conflict of Interest and Authorship Conformation Form

- All authors have participated in (a) conception and design, or analysis and interpretation of the data; (b) drafting the article or revising it critically for important intellectual content; and (c) approval of the final version.
- This manuscript has not been submitted to, nor is under review at, another journal or other publishing venue.
- The authors have no affiliation with any organization with a direct or indirect financial interest in the subject matter discussed in the manuscript
- The authors declare no conflict of interests.
